# Protein Hydrolysates Derived from Animals and Plants—A Review of Production Methods and Antioxidant Activity

**DOI:** 10.3390/foods11131953

**Published:** 2022-06-30

**Authors:** Michał Czelej, Katarzyna Garbacz, Tomasz Czernecki, Jacek Wawrzykowski, Adam Waśko

**Affiliations:** 1 Biolive Innovation Sp. z o. o., 3 Dobrzańskiego Street, 20-262 Lublin, Poland; katarzyna.garbacz@up.lublin.pl; 2 Department of Biotechnology, Microbiology and Human Nutrition, Faculty of Food Science and Biotechnology, University of Life Sciences in Lublin, 8 Skromna Street, 20-704 Lublin, Poland; tomasz.czernecki@up.lublin.pl (T.C.); adam.wasko@up.lublin.pl (A.W.); 3 Department of Biochemistry, Faculty of Veterinary Medicine, University of Life Sciences in Lublin, 12 Akademicka Street, 20-400 Lublin, Poland; jacek.wawrzykowski@up.lublin.pl

**Keywords:** bioactive peptides, protein hydrolysates, plants, animals, enzymatic hydrolysis, antioxidant activity

## Abstract

There is currently considerable interest on the use of animal, plant, and fungal sources in the production of bioactive peptides, as evidenced by the substantial body of research on the topic. Such sources provide cheap and environmentally friendly material as it often includes waste and by-products. Enzymatic hydrolysis is considered an efficient method of obtaining peptides capable of antioxidant activity. Those properties have been proven in terms of radical-scavenging capacity using the DPPH (1,1-diphenyl-2-picrylhydrazyl) and ABTS (2,2-azinobis-(3-ethyl-benzothiazoline-6-sulphonic acid)), hydroxyl and superoxide radical methods. Additionally, the reducing power, ferrous ion-chelating (FIC), ferric reducing antioxidant power (FRAP), and the ability of the protein hydrolysates to inhibit lipid peroxidation have also been explored. The results collected in this review clearly indicate that the substrate properties, as well as the conditions under which the hydrolysis reaction is carried out, affect the final antioxidant potential of the obtained peptides. This is mainly due to the structural properties of the obtained compounds such as size or amino acid sequences.

## 1. Introduction

With the currently evolving human consumption behavior, the demand for healthy and natural food products is on the increase [1]. At the same time, however, many synthetic antioxidants including butylated hydroxytoluene (BHT) butylated hydroxyanisole, tert-butylhydroquinone, and propyl gallate are frequently added to food to prevent its premature deterioration [2,3]. The longstanding consumption of these compounds has raised concerns associated with their potential hazardous effects on the human body [4,5]. Hence, identifying natural substitutes for synthetic antioxidants. Numerous scientific publications pay special attention to the use of animal, plant and fungal sources for the production of biologically active peptides and proteins, which in the future will be able to largely replace the current synthetic compounds on the market [6,7,8,9,10].

Bioactive peptides (BPs) are a group of organic compounds characterized by many health-promoting properties such us immunomodulatory, antihypertensive, antimicrobial, insulin-mimetic, or antioxidant effect. BPs can be obtained during gastrointestinal digestion, fermentation, enzymatic hydrolysis, or in chemical reactions in vitro [3,11,12]. Generally, peptides are formed of 2 to 100 amino acid residues. Their bioactive potency is dependent on the inherent composition and sequence of amino acid residues. Peptides encoded in the sequence of their parent proteins are mostly inactive, but they can be converted into their active form by applying enzymes or chemical reagents.

Enzymatic hydrolysis is considered a particularly important method of generating bio-functional peptides. Compared to chemical methods, it required mild reaction conditions, generates few undesirable side products, and facilitates high yield and product quality [13]. Additionally, the bioactivity of the obtained protein hydrolysates can be improved during this process as it is affected by several parameters, including the type of enzyme, processing time, pH, and temperature [14]. Various proteinases such as alcalase [1,3,5,15,16,17,18,19,20,21,22,23], neutrase [24,25], papain [1,17,26,27,28], pepsin [1,15,25,26,28,29,30,31,32,33,34,35,36], trypsin [1,20,21,25,28,29,32,33,34,35,36,37,38,39,40], chymotrypsin [28,29,40], protex 6L [37], seabzyme L 200 [37], viscozyme L [41], pancreatin [3,4,30,33,41,42,43,44], cellulase [45], thermolysin [21], flavourzyme [3,19,25,46], corolase [37,47,48], protamex [22,25,37,49], and bromelain [37,45,50,51,52,53,54,55,56] have been extensively employed in the production of peptides with antioxidative properties. Furthermore, biologically active peptides have also been obtained using protease of bacterial and fungal origin such as *Bacillus subtilis* A26 [51], *Bacillus licheniformis* NH1, *Bacillus pumilus* A1, *Bacillus mojavensis* A21, *Aspergillus clavatus* ES1, *Saccharomyces pastorianus* [24], and *Yarrowia lipolytica* [52].

In the last decade, antioxidative proteins and their derived hydrolysates have been subject to particular research scrutiny. They could constitute natural and safe alternatives to synthetic antioxidants as after oral administration, they are degraded into the harmless components of amino acids, as opposed to synthetic antioxidants that can be adsorbed and remain in the body for long periods of time and can eventually cause cancer [30].

Hence, the following paper focuses on various protein hydrolysates with shown antioxidative properties, derived from different kinds of animal, plant and fungal proteins and described in literature in recent years. To this end, we cite results relevant to a variety of sources, including one’s alternative to well-established material such as soybeans, wheat or whey. Although enzymatic hydrolysis has many advantages (e.g., the nature of GRAS) and is widely used to obtain protein hydrolysates, researchers are still looking for new approaches to increase the yield of bioactive peptides. Therefore, it is crucial in the conducted studies to use different enzymatic protocols that will allow the selection of optimal process conditions adapted to the protein source and its nature, which will increase the efficiency of the reaction by reducing the hydrolysis time, reduce the process cost and allow to obtain the desired peptides. Our aim was to collect recent achievements in this field and elaborate on the effectiveness of different enzymes against different protein sources. The results presented in the following article will help scientists to make more informed decisions regarding the choice of enzymes as well as optimization of the hydrolysis process in terms of the appropriate enzyme/substrate ratio, reaction time, pH, temperature, and deactivation conditions.

## 2. The Animal Kingdom

### 2.1. Methods of Obtaining Protein Hydrolysates of Animal Origin

A wide range of protein hydrolysates with antioxidative potential have been extracted from various fish, insects, domestic fowl, as well as zoonotic products (Table 1).

### 2.2. Antioxidant Activity of Protein Hydrolysates Derived from Fish

Blanco et al. [35] prepared and investigated four hydrolysates derived from fish skin collagen of two shark species (*Prionace glauca* and *Scyliorhinus canicula*) and the two bonny fish species (*Thunnus albacares* and *Xiphias gladius*). All of the samples were screened for antioxidant activity and radical-scavenging capacity utilizing DPPH (1,1-diphenyl-2-picrylhydrazyl) and ABTS (2,2-azino-bis-(3-ethyl-benzothiazoline-6-sulphonic acid)). The recorded results were expressed in milligrams of BHT equivalent per 1 mL of hydrolysate (mg BHT Eq mL^−1^). Furthermore, the inhibition of lipid peroxidation (estimated with a β-carotene assay) of collagen hydrolysates from these species was also evaluated. Previous studies [59,60] suggested that the antioxidant activity of protein hydrolysates was associated with the degree of hydrolysis. Blanco et al., observed the lowest antioxidant potency in the hydrolysate obtained from *Prionace glauca* (405.3 and 151.2 mg BHT Eq mL^−1^, respectively for the DPPH and ABTS method), which simultaneously exhibited the highest degree of hydrolysis (16.52%). Meanwhile, although the hydrolysate prepared from *Xiphias gladius* demonstrated a higher degree of hydrolysis (12.56%) then the one generated from *Thunnus albacores* (11.49%), it was identified as capable of the highest antioxidant activity (677.20 and 253.77 mg BHT Eq mL^−1^, respectively for the DPPH and ABTS assay). The findings suggest that the antioxidant activity of protein hydrolysates could be associated not only with the degree of hydrolysis, but also with other factors such as the presence of amino acids capable of interacting with free radicals, e.g., the SH group in cysteine [59,61,62]. On the basis on the β-carotene evaluation, the highest antioxidant potential was observed for the hydrolysate derived from *Scyliorhinus canicula* (20.86 mg BHT Eq mL^−1^) which displayed a high degree of hydrolysis (15.8%).

Ktari et al. [54] carried out research on the *Salaria basilica* protein hydrolysates obtained by subjecting fish material this to hydrolysis using crude alkaline protease extracts from the same species, sardinella, and smooth hound. The study revealed that rats fed with a hypercholesterolemic diet and treated with the obtained hydrolysate were protected against oxidative stress caused by their high-cholesterol diet. A dose of 400 mg of the protein hydrolysate per kg of body weight administrated for eight weeks was shown to restore the levels of enzymatic as well as non-enzymatic components of the antioxidant barrier. All three *Salaria basilica* hydrolysates were reported to trigger a decrease in the activity of superoxide dismutase (SOD) (by between 30 and 45 U/mg protein), glutathione peroxidase (GSH-Px) (between 0.0025 and 0.0035 µmol GSH mg protein/min), and catalase (between 3 and 4 μmol H_2_O_2_/mg protein/min) relative to the same parameters examined in the hearts of hypercholesterolemic rats (aprox. 58 U/mg protein, 0.0044 µmol GSH mg protein/min and 7.5 μmol H_2_O_2_/mg protein/min, respectively). In turn, the level of glutathione (GSH) was observed to increase in the heart of rats treated with the three obtained hydrolysates (by between 450 and 650 μg/g tissue) as compared to rats fed with a high-cholesterol diet (approx. 260 μg/g tissue). A similar effect of the same *Salaria basilica* hydrolysates was reported in the liver of rats with alloxan-induced diabetes [55]. These findings make the *Salaria basilica* protein hydrolysates promising antioxidants potentially beneficial in cases of diabetes and cardiovascular diseases caused by high-cholesterol levels.

Jiang et al. [1] described the preparation and antioxidant potency of five protein hydrolysates derived from *Decapterus maruadsi*. All of the obtained hydrolysates were subjected to scavenging activity (DPPH, O_2_·) and reducing power assays by way of redox-linked colorimetric reactions and electron-transfer evaluations, respectively. It was reported that the DPPH scavenging effect screen revealed an increase whose extent depended on the enzyme used: neutral (32.33%) < pepsin (32.63%) < alcalase (39.36%) < trypsin (39.37%) < papain (40.21%), whereas the scavenging capacity evaluated with the O_2_· method increased as follows: pepsin (7.57%) < trypsin (18.85%) < papain (20.78%) < alcalase (23.06%) < neutral (25.25%). Moreover, all five hydrolysates were reported to have reduced power (0.33, 0.309, 0.260, 0.234 and 0.229, respectively for the trypsin, alcalase, papain, neutral and pepsin hydrolysates). Given that alcalase was identified as an enzyme capable of generating bioactive peptides from natural protein hydrolysates [63] and based on the above-mentioned results, the researchers selected alcalase hydrolysate for further studies. After ultrafiltration, the fraction of alcalase hydrolysate with the molecular weight of under 5 kDa demonstrated the highest antioxidative activity (50.54 and 28.11% for the DPPH and O_2_· radical assays, respectively, 0.343 for reducing power estimation). Next, it was purified by way of gel filtration chromatography. Of the four obtained fractions, the one with the molecular weight ranging from 0.3 to 1.2 kDa showed the potential for the strongest activity and as such, it was further purified by way of two-fold reverse-phase high-performance liquid chromatography (RP-HPLC). Eventually, matrix-assisted laser desorption ionization time-of-flight/time-of-flight mass spectrometry (MALDI-TOF/TOF MS) allowed the identification of the purified peptides as His-Asp-His-Pro-Val-Cys (706.8 Da) and His-Glu-LysVal-Cys (614.7 Da), whose DPPH (0.0310%, 0.0677%; respectively) and O_2_· radical scavenging activity (0.3817%, 0.3744%; respectively) made them promising antioxidative components in functional foods.

Tejpal et al. [36] studied protein hydrolysates formed from *Oreochromis niloticus* by-products stored on ice for 0, 24 and 48 h. Antioxidant properties of these hydrolysates were tested in terms of DPPH free radical scavenging activity, ferric reducing antioxidant power (FRAP), and linoleic acid peroxidation inhibition activity (LAPI). It was demonstrated that pepsin hydrolysate obtained from the fresh waste sample was capable of stronger scavenging activity at the concentrations of 1 mg/mL (45.89%), 1.5 mg/mL (52.36%), 2 mg/mL (64.79%), and 2.5 mg/mL (73.62%), as well as higher ferric reducing antioxidant power at the concentrations of 20 mg/mL (0.7), 30 mg/mL (0.82), and 50 mg/mL (1.30), as compared to hydrolysates prepared from the ice storage waste. These findings are thought to result from differences in the sequence of peptides obtained from the fresh and the ice-stored by-products. The cited authors found that hydrolysis of the fresh fish waste released peptides that were bigger and contained more His, Arg, Tyr and Phe compared to those generated from hydrolysis of the ice stored fish waste. The presence of the above-mentioned amino acids was associated with stronger radical scavenging activity [64]. In addition, the hydrolysate produced from fresh *Oreochromis niloticus* by-products induced a higher lipid peroxidation inhibitory effect (55.49%) than the hydrolysate obtained after 24 (43.57%) and 48 (43.65%) hours of ice storage. These findings coincide with the report by Elavarasan et al. [65] who noted that protein hydrolysates can act as antioxidants towards lipid peroxidation. Furthermore, Roslan et al. [13] reported that alcalase hydrolysates prepared from *Oreochromis niloticus* possess the ability to inhibit the angiotensin I-converting enzyme (ACE) at 88.26%., which is of particular significance in regulating blood pressure.

Vieira et al. [24] investigated protein hydrolysates produced from *Sardina pilchardus* treated with brewer’s spent yeast proteases (Bsy), alcalase and neutrase. The DPPH radical-scavenging assay and ferric ion reducing antioxidant power (FRAP) assay were used to evaluate the antioxidant potency of the newly obtained hydrolysates. The results were presented as micromole of Trolox per mL of hydrolysate (µM TE mL^−1^). The angiotensin I-converting enzyme inhibitory (ACE-I) activity was also analyzed. The researchers demonstrated that the muscle hydrolysate prepared with neutrase and alcalase has over twice the radical-scavenging capacity (780, 870 µM TE mL^−1^, respectively) compared to the one produced using brewer’s spent yeast proteases (360 µM TE mL^−1^). Likewise, the ferric ion reducing antioxidant power was two times higher for muscle neutrase and alcalase hydrolysates (162, 160 µM TE mL^−1^, respectively) as compared to the muscle hydrolysate obtained with brewer´s spent yeast proteases (80 µM TE mL^−1^). It was also shown that the alcalase hydrolysate produced from the viscera of *Sardina pilchardus* showed the highest antioxidant activity screened using both the DPPH (840 µM TE mL^−1^) and FRAP (190 µM TE mL^−1^) methods, whereas the viscera neutrase hydrolysate revealed the lowest antioxidant potential (660 and 110 µM TE mL^−1^ for DPPH and FRAP assay, respectively). In turn, the strongest ACE-I activity was recognized for muscle and viscera hydrolysates prepared with brewer´s spent yeast proteases (984 and 828 μg protein mL^−1^, respectively).

Taheri et al. [66] prepared bioactive peptides—antioxidant and ACE inhibitors, from minced Kawakawa (*Euthynnus affinis*) muscle using pepsin extracted from skipjack tuna. The thus obtained hydrolysates were firstly divided into four fractions depending on the molecular weight, and then assessed using DPPH scavenging chelating metal ion assay (Fe^2+^), reducing power assay, and linoleic acid model system. It was concluded that the investigated fraction samples were good antioxidants whose radical scavenging was enhanced with increasing concentration of peptides in the test sample. The hydrolysates reached almost 100% of DPPH scavenging activity, a result comparable to a commercial BHT antioxidant. As for the other mentioned methods, the results were also promising: the percentage of metal chelating activity almost reached the value of EDTA. Peptide fractions revealed the lowest reductant power for the peptide fraction with the highest molecular weight, and no significant difference was observed for fractions, with ascorbic acid used as positive control. The results in terms of inhibition of the linoleic auto-oxidation showed that the fractions with lower molecular weight were the most potent and effective inhibitors, almost as good as vitamin E. An interesting proposal entailing food preservation with the use of antioxidant and antimicrobial peptides obtained from the Argentine croaker was presented in Romani et al. [67]. The authors studied the potential use of peptides bonded onto polyethylene film surfaces as actively antioxidant packaging materials. Peptides were obtained via enzymatic hydrolysis with alcalase as the proteolytic enzyme at the enzyme/protein ratio of 30 U/g. It was reported that clean native polyethylene film did not show any significant radical scavenging ability, whereas the polyethylene surface enriched with peptides reached 1 nmol Trolox/cm^2^ of free radical scavenging capacity. Currently, work is underway to increase the quantity of peptides bound to the surface as well as to test the ability to preserve food containing compounds contributing to food spoilage, including lipids, lycopene, and ascorbic acid.

Yaghoubzadeh et al. [68] studied the bioactivity of protein hydrolysates obtained from the skin of rainbow trout. The antioxidant properties of hydrolysates were evaluated by measuring the DPPH radical inhibitory power as well as the ferric reducing antioxidant ability. The cited article described two hydrolysis reactions preceded by deactivation of the internal enzymes present in the fish. The first reaction involved the addition of 1% of alcalase against the protein content, the second was performed using flavourzyme enzyme with the addition of 1% protein content. The increasing trend in terms of DPPH radical inhibition was strongly correlated with increasing concentrations of the tested hydrolysate samples in for reactions involving the addition of alcalase and flavourzyme. Furthermore, a slightly higher percentage of DPPH free radical scavenging was obtained in the case of the flavourzyme hydrolyzed product—at the concentration of 800 pm it reached almost 50%. The trend in terms of reducing power was similar: the higher the concentration of hydrolysate, the greater the reducing power. The highest reducing power was also observed for the flavourzyme enzyme.

### 2.3. Antioxidant Potency of Protein Hydrolysates Prepared from Insects

Zielińska et al. [44] extensively examined protein hydrolysates obtained from heat-treated edible insects: *Gryllodes sigillatus*, *Tenebrio molitor* and *Schistocerca gregaria*. For each of the species, the samples were divided into four groups: boiled (in boiling water for 10 min at 100 °C), baked (in a heated oven at 150 °C for 10 min), raw, and proteins derived from raw insects. All of the samples were digested with gastrointestinal enzymes: α-amylase, pepsin, pancreatin, and a bile extract solution. Next, the obtained hydrolysates were subjected to dialysis (using a membrane tube with molecular weight cut-off of 3.5 kDa) and an absorption process. DPPH and ABTS radical scavenging activity as well as ion chelating potency and reducing power were measured to estimate the antioxidant capacity of the respective hydrolysates. In addition, anti-inflammatory potency was analyzed in terms of lipoxygenase and cyclooxygenase-2 inhibitory potential. The results of these assays (with the exception of reducing power) were expressed as IC_50_ values, that is the peptide concentration required for 50% inhibition. It was found that the hydrolysate obtained from raw *T. molitor* was capable of producing the strongest ABTS antiradical activity (IC_50_ = 5.3 µg/mL) whereas the ones derived from baked *G. sigillatus* and the proteins from *S. gregaria* showed the highest DPPH radical scavenging potency (IC_50_ = 28.5 µg/mL in both cases) before the absorption process. In turn, after the absorption, the hydrolysate generated from the baked *G. sigillatus* was identified as the most potent in the ABTS (IC_50_ = 10.7 µg/mL) and DPPH (IC_50_ = 10.9 µg/mL) assays. On the other hand, the lowest values of ABTS and DPPH scavenging activity before the absorption were observed for the hydrolysates of boil and row *T. molitor* (IC_50_ = 28.9 and 109.4 µg/mL, respectively), whereas after the absorption process the lowest ABTS and DPPH evaluation values were noted for the hydrolysates formed from the protein of *T. molitor* (IC_50_ = 24.31 µg/mL) and *S. gregaria* (IC_50_ = 88.81 µg/mL). In terms of the Fe ^2+^ chelating capacity, the hydrolysate prepared from the protein of *G. sigillatus* was shown to be the most active (IC_50_ = 16.18 µg/mL) before the absorption, while the one generated from boiled *S. gregaria* exhibited the strongest metal chelating potency (IC_50_ = 5.57 µg/mL) after the absorption process. Furthermore, the hydrolysates derived from raw and baked *G. sigillatus* were reported as having the highest reducing power before (0.771) and after (0.286) absorption, respectively. The anti-inflammatory potential tests revealed that the hydrolysate obtained from the protein of *S. gregaria* was the most potent in inhibiting the activity of lipoxygenase (LOX) both before (IC_50_ = 0.65 µg/mL) and after (IC_50_ = 3.13 µg/mL) the process of absorption. Moreover, the hydrolysates from raw material and protein of *S. gregaria* showed the strongest inhibitory effect against cyclooxygenase (COX) before (IC_50_ = 10.91 µg/mL) and after (IC_50_ = 5.05 µg/mL) absorption, respectively.

Sousa et al. [69] studied protein hydrolysates obtained from edible lesser mealworms (*A. diaperinus*) and their potential antioxidant activity. The tested peptide samples were obtained by hydrolyzing with alcalase 2.5 L and corolase PP. In each case, the enzyme to substrate ratios were 0.5, 1.5 and 3.0% (*v*/*w*). DPPH and ORAC radical scavenging activity assays were employed to estimate the antioxidant capacity for the best peptide samples. The authors identified peptide mixture obtained through the longest hydrolysis process and with the highest E/S ratio as the most suitable antioxidant. It was strongly connected with increasing the degree of hydrolysis. In this case, the Trolox equivalent antioxidant capacity (TEAC) was 5.8 μmol TE/mL for alcalasee and 6.6 μmol TE/mL for corolse PP. The authors also considered ORAC values. Here, the results for the 1.5 and 3.0% E:S alcalase 2.5 L products were not significantly different. The results presented for corolase PP indicated the conditions of 3.0% (E/S) and 6 h-long reactions as the most suitable for obtaining the highest TEAC value (12.3 μmol TE/mL).

The biological potency of protein hydrolysate obtained from silkworm pupae was examined by Zhang et al. [70]. The hydrolysate was obtained from a 3-h reaction with the addition of 3% neutral protease and purified through ultrafiltration using a membrane with the molecular mass cut off sizes of 5 and 3 kDa. The lyophilized fractions were tested to determine the ABTS radical scavenging activity. During the studies, the authors identified and characterized two novel antioxidant peptides with the following sequences: FKGPACA and SVLGTGC. The peptides possessed high radical scavenging potential comparable with the results reported for such antioxidants as GSH and Trolox. The antioxidant effects can be due to the peptides’ short amino acid sequences. To determine the exact antioxidant fragments in their chains, the authors divided the peptides into shorter sections: FKGPACA into FKGP and ACA, and SVLGTGC into SVLG and TGC. They found that ACA and TGC fragments showed higher antioxidant activity than FKGP and SVLG. For this reason, the authors identified those amino acid fragments as the active centers responsible for antioxidation.

### 2.4. Antioxidant Capacity of Protein Hydrolysates Obtained from Domestic Fowl

Wang and Shahidi [46] investigated the antioxidative capacity of protein hydrolysates derived from turkey (*Meleagris gallopavo*) and chicken (*Gallus gallus domesticus*) meat. The researchers obtained the hydrolysates from an enzymatic hydrolysis reaction with flavourzyme. The obtained protein hydrolysates were subjected to ABTS, DPPH, and hydroxyl radical scavenging activity estimations as well as reducing power and ferrous ion chelating capacity assays with a view to evaluating their antioxidant potency. Both investigated hydrolysates were shown to have comparable antioxidative potential. There were no significant differences in the scavenging activity evaluated in terms of ABTS (turkey: 79.15%; chicken: 78.23%), DPPH (turkey: 88.33%; chicken: 86.52%), and hydroxyl radicals (turkey: 42.52%; chicken: 43.11%) between the analyzed hydrolysates. Likewise, the ferrous ion chelating capacity values were similar: 15.09% for the protein hydrolysate generated from turkey and 15.42% for the one prepared from chicken. Furthermore, the turkey and chicken hydrolysates exhibited similar reducing power, (0.89) (0.8), respectively.

### 2.5. Antioxidant Potential of Protein Hydrolysates Produced from Zoonotic Products

In a recent study, Al-Shamsi et al. [17] reported the in vitro antioxidant activity of camel (*Camelus dromedarius*) milk protein hydrolysates. The hydrolysates were obtained by reacting raw milk with three proteolytic enzymes: alcalase, bromelain and papain. The antioxidant capacity was assessed in terms of ABTS and DPPH radical scavenging activity as well as ferric-reducing antioxidant power (FRAP) and ferrous iron-chelating potency. The results of the evaluations (with the exception of the chelating activity which was calculated as a percentage) were presented as micromoles of Trolox equivalents (TE) per gram of the sample (µmol of TE/g). The DPPH method showed similar activity for bromelain (about 23 µmol of TE/g), papain (about 21 µmol of TE/g) and alcalase (about 19 µmol of TE/g) hydrolysates. In turn, the ABTS scavenging capacity of the hydrolysate produced with papain was two times higher (about 48 µmol of TE/g) than in the case of bromelain (about 20 µmol of TE/g), whereas the alcalase-produced hydrolysate showed moderate activity (about 34 µmol of TE/g). The reducing power of all three hydrolysates ranged between 30 and 40 µmol of TE/g. Similarly, the metal chelating activity was within the range from 30 to 40% for all studied hydrolysates. Further efforts were undertaken by Al-Shamsi et al. to investigate lipid peroxidation inhibitory potency of camel milk hydrolysates in two real food model systems i.e., grape seed oil-in-water emulsion and fish mince. The ability to prevent lipid peroxidation was studied by estimating thiobarbituric acid reactive substances assay. The hydrolysate generated with papain was found to have stronger potency in inhibiting lipid peroxidation when compared to hydrolysates prepared with alcalase and bromelain. Moreover, the study demonstrated that camel milk hydrolysates were more efficient in preventing TBARS formation at higher concentrations, rather than lower. Finally, camel milk without hydrolysis was not capable of inhibiting lipid peroxidation in the oil-in-water emulsion and fish mince systems.

Pokora et al. [52] evaluated the antioxidant capacity of protein hydrolysate and peptide fractions obtained from egg white. This hydrolysate was obtained using non-commercial serine protease from *Yarrowia lipolytica* yeast. Subsequently, it was ultra-filtered through a 3 kDa membrane and then fractionated with RP-HPLC. The hydrolysate and all of the produced fractions were evaluated in terms of antioxidant potency by analyzing their DPPH free radical scavenging activity, ferric reducing antioxidant power (FRAP), and Fe (II) ion chelation activity. The cited researchers demonstrated that the egg white hydrolysate showed strong ability to chelate Fe (II) with the value of 688.5 μg Fe^2+^/mg, high ferric reducing antioxidant capacity with the value of 35.3 μg Fe^2+^/mg, and noticeable free radical scavenging capacity with the value of 0.21 μM Trolox_eq_/mg. Furthermore, all of the prepared peptide fractions also demonstrated antioxidative potential within the ranges of 0.02–0.16 μM Trolox_eq_/mg, 2.09–12.48 μg Fe^2+^/mg and 21.73–206.96 μg Fe^2+^/mg for the DPPH, FRAP and Fe (II) chelating assays, respectively. Nonetheless, the antioxidative capacity of all of the peptide fractions was lower than that of the initial hydrolysate. Similar findings were reported by Li et al. [71]. The researchers observed that in some cases, purification of hydrolysates actually decreases their antioxidant activity, which is often due to an entire system of interactions between peptides and free amino acids. Additionally, Pokora et al. studied the cytokine-inducing potential of egg white protein hydrolysates and peptide fractions. The cytokine-inducing activity was examined under an ex vivo stimulation of human whole blood cell cultures containing lipopolysaccharide from *E. coli* and the tested hydrolysates. The results revealed that the egg white protein hydrolysate dosed at 100 µg/mL could stimulate the blood cells to release anti-inflammatory IL-10 in amounts comparable to those reported for lipopolysaccharide. At the same time, it was observed that the level of pro-inflammatory IL-6 generated in response to the investigated hydrolysate was noticeably higher in comparison to the untreated cells, but almost 50% lower than reported for lipopolysaccharide. Overall, the egg white protein hydrolysate appeared to be a good stimulator of cytokine production in human whole blood cell cultures.

## 3. The Plants Kingdom

### 3.1. Methods of Obtaining Protein Hydrolysates of Plant Origin

**Figure 1:**
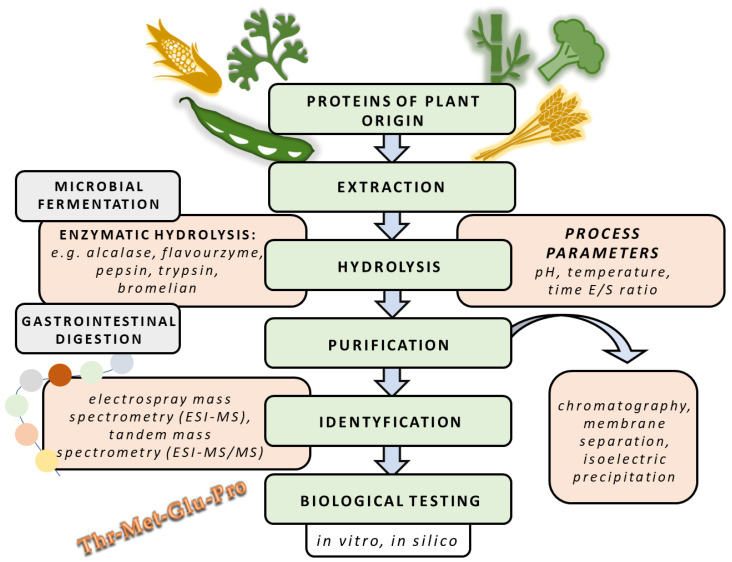
Diagram of bioactive peptide preparation from plant origin.

Given the fact that diet containing a significant number of fruits and vegetables is linked to lower incidence of diseases related to oxidative stress [39], many researchers have focused their investigations on antioxidative protein hydrolysates derived from various available plants (Table 2). Figure 1 shows a diagram of the extraction, purification and identification of bioactive peptides of plant origin.

### 3.2. Antioxidant Activity of Protein Hydrolysates Derived from Marine Plants

Paiva et al. [45] described the preparation and results of the antioxidant studies on peptide fractions derived from the *Fucus spirali* protein hydrolysate. After a two-step hydrolysis, first with cellulose then with bromelain, the obtained hydrolysate was subjected to ultrafiltration using three different membranes with the molecular weight cut-off of 1 and 3 kDa. This allowed the cited authors to prepare peptide fractions with different molecular weight ranges: Fr1 (<1 kDa), Fr2 (1 kDa ≤ Fr2 < 3 kDa), and Fr3 (≥3 kDa). The antioxidant activity of the above-mentioned fractions was tested using three assays: DPPH free radical scavenging activity (FRSA), ferrous ion-chelating (FIC), and ferric reducing antioxidant power (FRAP). The highest scavenging capacity in terms of DPPH free radicals was observed for Fr1 (86.03%) followed closely by Fr3 (80.50%), while the lowest result was obtained for Fr2 (32.73%). It is noteworthy that Fr1 showed radical scavenging potency comparable to that of synthetic antioxidant BHT (about 95%). In terms of FIC, Fr3 showed the strongest chelating capacity (47.33%), followed by Fr1 (40.28%) the Fr2 (28.47%). Similarly, Fr3 was found to exhibit the highest FRAP (0.538) when compared to Fr1 (0.334) and Fr2 (0.185). Moreover, Fr3 was shown to possess a remarkable ACE-inhibitory activity (86.85%).

Harnedy et al. [47] investigated protein hydrolysate produced from *Palmaria palmate* by way of digestion with food-grade Corolase^®^ PP enzyme.

The antioxidant activity of the newly obtained hydrolysate was screened in terms of oxygen radical absorbance capacity (ORAC) and ferric reducing antioxidant power (FRAP). The results of the above assays were presented as micromole of Trolox equivalents per gram dry weight (μmol TE/g dw). The authors found that the protein hydrolysate derived from *Palmaria palmate* showed antioxidant potency in both the ORAC (483.69 μmol TE/g dw) and the FRAP (25.29 μmol TE/g dw) evaluations. The studied hydrolysate was subsequently fractionated, after which the researchers identified, synthetized and examined the obtained peptides. one of the same, the peptide with the sequence: Ser-Asp-Ile-Thr-Arg-Pro-Gly-Gly-Asn-Met was observed to show the highest antioxidant activity (152.43 and 21.23 nmol TE/μmol peptide for ORAC and FRAP assays, respectively).

The biological potency of protein hydrolysate prepared from *Schizochytrium* sp. was investigated by Cai et al. [19]. This hydrolysate was obtained in a two-step hydrolysis, first with alcalase then with flavourzyme. Subsequently, it was divided into two fractions using a 3 kDa membrane. The antioxidant activity of *Schizochytrium* sp. protein hydrolysate was tested by conducting ABTS and DPPH radical scavenging examinations as well as reducing power evaluation. The researchers identified the fraction of hydrolysate characterized by molecular weight of under 3 kDa as possessing strong ABTS and DPPH scavenging capacity (IC_50_ = 17.5 and 350 µg/mL, respectively). Likewise, at the concentration of 1 mg/mL, higher reducing power was observed for the fraction containing peptides with molecular weight below 3 kDa, as compared to the fraction including peptides with molecular weight above 3 kDa (0.54 and 0.33 respectively). Furthermore, the antioxidant potential of the fraction with molecular weight below 3 kDa was examined in vivo in mice with acute alcohol-induced liver injury. The study demonstrated that mice treated with the *Schizochytrium* sp. protein hydrolysate dosed at 100 and 300 mg per kg of body weight for 24 consecutive were able to restore the activity of hepatic catalase (CAT), SOD and GSH-Px as well as the level of glutathione (GSH). In addition, the fraction containing *Schizochytrium* sp. peptides with molecular weight below 3 kDa was shown to induce a decrease in the activity of serum alanine aminotransferase (ALT) (by 38.9% and 41.4%, respectively, for the doses of 100 and 300 mg kg^−1^ b.w.), aspartate aminotransferase (AST) (by 23.8% and 25.8%, respectively, at100 and 300 mg kg^−1^ b.w.), and the level of hepatic malondialdehyde MDA (by 27.0% and 38.7%, respectively, at 100 and 300 mg kg^−1^ b.w.) in comparison to group receiving only alcohol. The above findings evidence that *Schizochytrium* sp. protein hydrolysate is a promising antioxidant in the context of alcohol-induced liver diseases.

### 3.3. Antioxidant Capacity of Protein Hydrolysates Obtained from Cucurbitaceae

Dash and Ghosh [39] studied protein hydrolysates obtained from *Cucurbitaceae* seeds. The antioxidant potency of trypsin hydrolysates of *Cucurbit moschata*, *Citrullus lanatus* and *Lagenaria siceraria* was tested using DPPH, ABTS, hydrogen peroxide, and nitric oxide evaluations, as well as lipid peroxidation inhibition estimation assays. The results in terms of scavenging activity were presented as the IC_50_ value, that is, the concentration of sample required to scavenge 50% of free radicals. The cited authors found that the hydrolysates prepared from *L. siceraria* and *C. moschata* demonstrated radical-scavenging capacity over two times stronger (46 and 49.3 µg/mL, respectively) than that observed for *C. lanatus* (80.3 µg/mL) in the DPPH method. The ABTS assay has also revealed the highest radical scavenging for the *L. siceraria* hydrolysate (108 µg/mL), followed by the hydrolysate *C. moschata* (142.3 µg/mL), and from *C. lanatus* (179 µg/mL). It was reported that the nitric oxide scavenging potency of the examined hydrolysates increased in the following order: *C. lanatus* (102 µg/mL) < *C. moschata* (59.6 µg/mL) < *L. siceraria* (32.6 µg/mL). Similarly, the inhibition of hydrogen peroxide radicals by the studied hydrolysates increased in the following sequence: *C. lanatus* (243.3 µg/mL) < *C. moschata* (153.6 µg/mL) < *L. siceraria* (141.6 µg/mL). Moreover, the hydrolysate formed from *Lagenaria siceraria* was identified as capable of lipid peroxidation inhibitory activity that was two times higher (20 µg/mL) than observed for *Cucurbit moschata* (43.6 µg/mL) and *Citrullus lanatus* (49.6 µg/mL).

### 3.4. Antioxidant Potential of Protein Hydrolysates Produced from Vigna radiata

Gupta et al. [20] investigated the biological properties of mungbean (*Vigna radiata*) vicilin protein hydrolysate. The antioxidant potency of alcalase and trypsin-generated mungbean vicilin protein hydrolysates was screened in terms of DPPH and ABTS radical scavenging potential, as well as ferric-reducing antioxidant power, and ferrous ion chelating potency. Furthermore, the antiproliferative effects on human breast cancer cells and ACE inhibitory activity of the hydrolysates were also examined. The research indicated that alcalase mungbean vicilin protein hydrolysate showed stronger DPPH and ABTS scavenging capacity (IC_50_ = 0.77 and 0.78 µg/mL, respectively) than its trypsin counterpart (IC_50_ about 1.3 µg/mL for both evaluations). Likewise, higher metal chelating activity was observed for the alcalase (about 70%) as compared to the trypsin (about 40%) hydrolysate. On the other hand, the ferric-reducing antioxidant power and reducing power assay results were lower for alcalase hydrolysate (about 290 mmol Fe (II)/mg extract and about 0.45%, respectively) as compared to the trypsin analogue (about 340 mmol Fe(II)/mg extract and about 0.55%, respectively). However, in terms of ACE inhibitory effects, the alcalase mungbean vicilin protein hydrolysate was found to be more active (IC_50_ = 0.32 mg/mL) relative to trypsin mungbean vicilin protein hydrolysate (IC_50_ = 0.54 mg/mL). The antiproliferative effects of *Vigna radiate* protein hydrolysate were investigated against two breast cancer cell lines: MCF-7 and MDA-MB-231. The mungbean seed protein hydrolysate prepared using trypsin was found to be more cytotoxic towards MCF-7 cells (IC_50_ = 0.45 mg/mL) than the mungbean seed protein hydrolysate produced using alcalase (IC_50_ = 0.73 mg/mL). In turn, the alcalase hydrolysate of mungbean vicilin was more cytotoxic against MDA-MB-231 (IC_50_ = 0.48 mg/mL) as compared to the analogue obtained with trypsin (IC_50_ = 0.54 mg/mL).

Mung bean peptides were also investigated by Xie et al. [79]. The authors subjected dehulled and ground beans to two-hour enzymatic reactions carried out using: alcalase, neutrase, papain and protamex, with the enzyme: substrate ratio of 3:100 (*w*/*v*). All of the reactions were ended with inactivation of the enzyme by boiling. Next, the obtained peptide solutions were centrifuged, adjusted to pH 4.2, dialyzed, and lyophilized. Before determining the antioxidation capacity, the prepared powders were subjected to DH determination. Of the four enzymes, the authors selected alcalase for further study due to its achievement of the highest degree of hydrolysis. Before the antioxidant assays, the lyophilized powder was dissolved and subjected to ultrafiltration to create three permeates with different molecular weights: MPBHs-I (<3 kDa), MPBH-s-II (3–10 kDa), and MPBHs-III (>10 kDa). The DPPH scavenging capacity for the <3 kDa solution was the highest and almost as high as for the ascorbic acid control sample (74% radical scavenging). Moreover, in terms of the hydroxyl radical scavenging ability, MPBHs-I produced the highest value of circa 50%. Similarly, in the case of Fe^2+^ chelating activity and reducing power, this fraction returned the best results ability. However, the values were nonetheless significantly lower than for the tested ascorbic acid sample.

### 3.5. Antioxidant Sctivity of Protein Hydrolysates Generated from Zingiberaceae

Inthuwanarud et al. [30] prepared and studied fifteen pepsin/pancreatin hydrolysates derived from plants of the *Zingiberaceae* family. All of the samples were examined in terms of their in vitro antioxidant activity using the DPPH method. The obtained results were expressed as IC_50_ value. Ascorbic acid was used for reference in the comparison of the IC_50_ values of the hydrolysates. Among the fifteen selected plant species, protein hydrolysates obtained from four of the same (*Alpinia galangal*, *Curcuma longa*, *Kaempferia pafiflora* and *Zingiber officinale)* exhibited no antioxidant capacity. As for the remaining eleven protein hydrolysates formed from *Zingiberaceae* plants, the observed antioxidant potency increased in the following sequence: *Boesenbergia pandurata* (IC_50_ = 131.8 µg/mL) < *Curcuma amarissima* (IC_50_ = 127.0 µg/mL) < *Curcuma aromatic* (IC_50_ = 118.2 µg/mL) < *Curcuma aeruginosa* (IC_50_ = 84.5 µg/mL) < *Hedychium coronarium* (IC_50_ = 56.1 µg/mL) < *Zingiber zerumbet* (IC_50_ = 54.6 µg/mL) < *Zingiber ottensii* (IC_50_ = 44.9 µg/mL) < *Curcuma xanthorrhiza* (IC_50_ = 39.4 µg/mL) < *Zingiber cassumunar* (IC_50_ = 38.6 µg/mL) < *Curcuma sp*. (IC_50_ = 36.3 µg/mL) < *Curcuma zedoaria* (IC_50_ = 25.7 µg/mL). It should be noted that the strongest antioxidant activity, observed for the protein hydrolysate obtained from *Curcuma zedoaria,* was very close to that of ascorbic acid (IC_50_ = 22.3 µg/mL). Therefore, this protein hydrolysate was chosen by the authors for further purification and examination. Of the five fractions obtained from the Q Sepharose ion exchange chromatography, only the one eluting in the 0.5M NaCl showed antioxidant capacity with the IC_50_ = 41.78 µg/mL. The same fraction was also found to be capable of inducing lower cytotoxic effects towards the human tumor derived hepatoma (HEP-G2) and colon (SW620) cell lines (IC_50_ = 200.8 and 241.0 µg/mL, respectively). Moreover, the MALDI-TOF analysis determined the molecular weight of the active protein hydrolysate as approximately 12.4–12.8 kD.

### 3.6. Antioxidant Potency of Protein Hydrolysates Formed from Moringa oleifera

Garza et al. [40] evaluated the antioxidant capacity of *Moringa oleifera* protein hydrolysates and peptide fractions. Protein hydrolysates from the seeds of the above-mentioned tree were obtained by way of 2.5 and 5-h enzymatic hydrolysis with three proteolytic enzymes: pepsin, trypsin, and chymotrypsin. Next, the newly obtained hydrolysates were subjected to ultrafiltration using a 10 kDa membrane in order to prepare peptide fractions. The antioxidant potency of the protein hydrolysates as well as the respective peptide fractions was evaluated using the ABTS assay and the results were presented as mM Equivalents of Trolox (ET) per mg of protein. Based on the performed examination, the authors found that peptide fractions > 10 kDa digested for 5 h were capable of inducing stronger antioxidant activity as compared to those digested for 2.5 h as well as the protein hydrolysates. The antioxidant activity of hydrolysates generated with trypsin (4.19 mM ET/mg protein for 2.5 h and 5.20 mM ET/mg protein for 5 h) was reported to be comparable to the results observed for chymotrypsin-formed hydrolysates (5.22 mM ET/mg protein for 2.5 h and 6.02 mM ET/mg protein for 5 h). In turn, the hydrolysate obtained with the mixture of pepsin and trypsin in 5-h hydrolysis was shown to be capable the highest antioxidant potency (13.86 mM ET/mg protein) of all of the protein hydrolysates, but the result was nonetheless lower than that for the peptide fraction formed by chymotrypsin for 5 h (17.86 mM ET/mg protein) or the mixture of pepsin and trypsin in 5 and 2.5-h hydrolysis (18.26 and 27.43 mM ET/mg protein, respectively). The results of this study, which are in line with the report of Zhao et al. [80], evidence that the antioxidant activity of protein hydrolysates is dependent on the specificity of the proteolytic enzyme and the duration of hydrolysis.

## 4. The Fungus Kingdom

Kimatu et al. [3] carried out a study on *Agaricus bisporus* protein hydrolysates. The hydrolysates were obtained from the reaction of enzymatic hydrolysis with three different enzymes: alcalase, pancreatin, flavourzyme and two mixtures: alcalase with pancreatin and alcalase with flavourzyme. Next, each hydrolysate was ultrafiltered using four different membranes with the molecular weight cut-off of 1, 3, 5 and 10 kDa. All of the obtained hydrolysates and peptide fractions were screened for antioxidant activity in terms of DPPH radical scavenging activity (DRSA), metal chelating potency, and ferric reducing antioxidant power (FRAP). The results of the DRSA and Fe ^2+^ chelating capacity analysis were expressed as EC_50_ values, i.e., the sample concentration of required for a 50% reduction in DRSA and the sample concentration that reduced chelating activity by 50%. The study revealed that of all of the studied hydrolysates, the one prepared with pancreatin was capable of demonstrating the highest radical scavenging activity (EC_50_ = 0.25 mg/mL). The peptide fractions < 1 kDa and 3–5 kDa were found to exhibit the least scavenging potency with respect to their parent hydrolysates and other peptide fractions. In turn, the peptide fraction in the range of 1–3 kDa obtained by treatment with pancreatin showed the strongest DRSA activity (EC_50_ = 0.13 mg/mL). In terms of ferric reducing antioxidant power, all of the protein hydrolysates and peptide fractions were observed to show a concentration-dependent increase. Likewise, the highest FRAP was reported for pancreatin hydrolysate (0.16 and 0.41 at the concentration of 1 and 3 mg/mL, respectively), and its peptide fraction 1–3 kDa (0.62 at the concentration of 3 mg/mL). On the other hand, the alcalase hydrolysate was confirmed as having the strongest capacity to chelate Fe^2+^ (EC_50_ = 0.96 mg/mL) relative to other hydrolysates. The alcalase peptide fraction 1–3 kDa was also shown to induce the highest metal chelating activity (EC_50_ = 1.2 mg/mL) when compared to other peptide fractions.

## 5. Structure—Activity Relationship

The antioxidant activity of protein hydrolysates and peptides is influenced both by the methods with which they are obtained, as well as their structural features. Therefore, scientists in their research pay more and more attention to the relationship between the structure of peptides and their activity (SAR). Particle size, amino acid composition and sequence, as well as hydrophobicity are mainly analyzed. However, still these relationships are not fully clear [81]. This is due to the fact that several factors interact simultaneously, e.g., hydrolysis conditions, size and amino acid composition of the obtained peptide, which ultimately determines its final activity. When discussing the influence of peptide size on the antioxidant potential, the authors of numerous publications point out that the peptides with smaller mass (≤3 kDa) are characterized by higher antioxidant activity. In the presented literature review, similar results were noticed, among others, analyzing the work of Phonghtai et al., 2018; Cai et al., 2017, who obtained the highest antioxidant activity for peptide fractions < 3 kDa. From the literature collected in this review, we can clearly see the differences in the molecular weight profiles of the hydrolysates. This is related to the specificity of the substrates as well as the activity of the enzymes studied under the given reaction conditions. Obtaining peptides with lower molecular weight is critically affected by the hydrolysis step—its time, the enzymes used or the temperature. Yu et al. (2018) in their work performed hydrolysis of *Ruditapes philippinarum* using trypsin, neutrase and pepsin. The neutrase hydrolysate contained the highest content of low molecular weight peptides. This is probably due to the fact that neutrase is an endopeptidase that does not have a specific enzymatic cutting site.

Another widely discussed aspect in the literature is the effect of amino acid composition of peptides on their activity. Aromatic amino acids (Tyr; His; Trp; Phe) show high free radical scavenging ability. This is due to the fact that they can transfer protons to electron-deficient radicals. One of the most widely discussed amino acids in the literature in the context of antioxidant activity is histidine. Histidine has an imidazole ring in its structure, which is responsible for scavenging lipid peroxyl radicals or chelating metal ions [6]. Peptides with antioxidant activity containing histidine in their composition have been discussed by Ahn et al., 2014; Homayouni-Tabrizi et al., 2017; Shazly et al., 2017; Jemil et al., 2017; Jiang et al., 2014, among others. Some authors also point out that the position of histidine on the N-terminus of the amino acid chain increases its bioactivity. Peptides containing hydrophobic amino acids, i.e., Ala, Tyr, Pro, Phe, Ile, Leu, and Phy, Trp, as well as peptides containing cysteine in their chains are also characterized by high antioxidant activity. The future of biological peptide activity analysis is quantitative structure-activity relationship modeling (QSAR). The multidimensional data obtained in this method allow to understand many aspects of chemical and biological interactions. Moreover, it reduces the costs associated with classical analytical methods.

## 6. Conclusions and Future Perspective

Biologically active peptides, due to their antioxidant, immunomodulatory, anticancer and antimicrobial properties, can be successfully used to create functional foods. Additionally, some of them are characterized by high specificity, lack of toxicity and high efficiency at low doses. Despite the above advantages, the use of BPs in functional foods is still limited. To change this, further research is needed on the extraction of the peptides, the stability of their biological activity, and the determination of the relationship between their structure and activity. According to available study, biologically active protein hydrolysates could constitute a valuable natural component for future applications not only in food products but also in the pharmaceutical, nutraceutical, and cosmetic industries. As demonstrated in this review, various protein sources subjected to various treatments can produce peptide mixtures with different antioxidant properties. Nearly all of above examples of protein sources, be it animal, plant, or fungal, can be used to obtain a peptide mixture with significant antioxidant properties. As clearly demonstrated in the above article, the radical scavenging capacity is primarily dependent on hydrolysis conditions such as: enzyme type, E:S ratio, temperature, and pH. In addition, the size of the extracted peptide and their amino acid sequence are also important. Therefore, future research on bioactive peptides will see strong development of both analytical methods and tools for in silico studies. Another challenge will be to determine the molecular mechanisms of action and bioavailability of peptides in a food matrix.

## Figures and Tables

**Table 1:** Methods of obtaining protein hydrolysates of animal origin.

Source	Part of Source	Enzymatic Hydrolysis						Enzymatic Deactivation				Dehydration	MW of PH/ Peptides Sequence	Antioxidant Activity PH/Peptide	References
		Type of Enzyme	E/S Ratio	pH	Temp (°C)	Time (h)	DH (%)	Temp (°C)	Time (min)	pH	Reagent				
*Ruditapes philippinarum*	whole	trypsin	3:100	9	45	4	17.54	100	10	-	-	lyophilization	Highest content fraction of MW 1–3 kDa	DPPH 38.77%	Yu et al., 2018
	whole	neutrase	3:100	6.5	45	4	18.3	100	10	-	-	lyophilization	Highest content fraction of MW <1 kDa	DPPH 68.55%	
	whole	pepsin	3:100	4	45	4	8.34	100	10	-	-	lyophilization	highest content of MW fraction >5 kDa.	DPPH 63.98%	
*Prionace glauca*	skin	pepsin	1:20	8	55	3	16.52	90	5	-	-	lyophilization	MW: 3 kDa	DPPH 422.97; 416.03 (mg BHT Eq/mL)—retentates (R) and permeates (P) fraction	Blanco et al., 2017
*Scyliorhinus canicula*	skin	pepsin	1:20	8	55	3	15.80	90	5	-	-	lyophilization	MW: 3 kDa	DPPH 603.40; 601.70 (mg BHT Eq/mL)—retentates (R) and permeates (P) fraction	
*Xiphias gladius*	skin	pepsin	1:20	8	55	3	12.56	90	5	-	-	lyophilization	MW: 3 kDa	DPPH 465.63; 448.0 (mg BHT Eq/mL)—retentates (R) and permeates (P) fraction	
*Thunnus albacores*	skin	pepsin	1:20	8	55	3	11.49	90	5	-	-	lyophilization	MW: 3 kDa	DPPH 435.97; 457.67 (mg BHT Eq/mL)—retentates (R) and permeates (P) fraction	
*Decapterus maruadsi*	muscle	alcalase	1:100	9.5	50	5	-	100	15	-	-	lyophilization	MW III: <5 kDa MW II: 5–10 kDa MW I: >10 kDa HEKVC HDHPVC	DPPH 39.36% —for PH I: 47.23% II: 36.27 III: 50.54 EC_50_ 0.0677 (mM) EC_50_ 0.0310 (mM)	Jiang et al., 2014
	muscle	neutral protease	1:100	7	50	5	-	100	15	-	-	lyophilization	n. d.	32.33% —for PH	
	muscle	papain	1:100	7	55	5	-	100	15	-	-	lyophilization	n. d.	40.21% —for PH	
	muscle	pepsin	1:100	2	37.5	5	-	100	15	-	-	lyophilization	n. d.	32.63% —for pH	
	muscle	trypsin	1:100	7.8	37.5	5	-	100	15	-	-	lyophilization	n. d.	39.37% —for PH	
*Oreochromis niloticus*	head, skin, trimmings, fins, frames and viscera	pepsin	1:100	2.5	37	3	-	100	15	-	-	spray drying	MW < 18 kDa	2.5 mg/mL DPPH scavenning 73.62%	Tejpal et al., 2017
*Sardinella aurita*	muscle	proteases from *B. subtilis* A26	3:1	8	45	5	10	-	-	-	0.1 N HCl	lyophilization	LVHYAGTVDYN; FRPQQPYPQP LEGDLKLSQE; NVLSGGTTMYPG; VIVEGELERT	highest reducing power and ORAC value	Jemil et al., 2017
*Rastrelliger kanagurta*	backbones	pepsin	1:100	2	37	6	24.7	100	10	-	-	lyophilization	n. d.	DPPH 46% ABTS 58.5%	Sheriff et al., 2017
	backbones	papain	1:100	6	37	3	18.1	100	10	-	-	lyophilization	n. d.	DPPH 36% ABTS 37.54%	
* Navodon septentrionalis *	head	papain	3:20	7.0	50	5	-	90	10	-	-	lyophilization	WEGPLK; GPP; GVPLT	DPPH IC_50_ 4.43 mg/mL IC_50_ 1.92 mg/mL IC_50_ 4.54 mg/mL	Chi et al., 2015
*Salmon*	backbones	Corolase^®^ PP	1:10	-	50	2	22.1	˃90	5	-	-	lyophilization	MW > 2.5 kDa	DPPH 32% *	Slizyte et al., 2016
	backbones	Corolase^®^ 7089	1:10	-	50	2	18.3	˃90	5	-	-	lyophilization	MW 2.5–6.5 kDa	DPPH 25%	
	backbones	Protamex^®^	1:10	-	50	2	20.9	˃90	5	-	-	lyophilization	MW 0.14 kDa—free amino acids	DPPH 38% *	
	backbones	Bromelain 400 GDU/g/Papain 100 TU/mg	1:10	-	50	2	16.8	˃90	5	-	-	lyophilization	MW 5.5–6.5 kDa	DPPH about 27% *	
	backbones	Protex 6L	1:10	-	50	2	18.2	˃90	5	-	-	lyophilization	MW 2.5–6.5 kDa	DPPH 25–26% *	
	backbones	Seabzyme L 200	1:10	-	50	2	17.1	˃90	5	-	-	lyophilization	MW 5.5–6.5 Da	DPPH 25–26% *	
	backbones	trypsin	1:10	-	50	2	18.1	˃90	5	-	-	lyophilization	MW > 2.5 kDa	DPPH about 21% *	
*Salmon*	pectoral fin	alcalase	1:100	7	50	8	around 10	100	15	-	-	lyophilization	n. d.	DPPH IC_50_ 4.76 mg/mL	Ahn et al., 2014
	pectoral fin	flavourzyme	1:100	7	50	8	around 10	100	15	-	-	lyophilization	n. d.	DPPH IC_50_ 3.62 mg/mL	
	pectoral fin	neutrase	1:100	7	50	8	around 10	100	15	-	-	lyophilization	n. d.	DPPH IC_50_ 4.95 mg/mL	
	pectoral fin	pepsin	1:100	2	37	8	around 10	100	15	-	-	lyophilization	Phe-Leu-ASN-Glu-Phe-Leu-His-Val (MW 1018.48 Da)	DPPH IC_50_ 1.63 mg/Ml—for preoteolytic hydrolysate DPPH IC_50_ 486 uM—for octapeptide	
	pectoral fin	Protamex	1:100	77	50	8	around 10	100	15	-	-	lyophilization	n.d.	DPPH IC_50_ 4.08 mg/mL	
	pectoral fin	trypsin	1:100		37	8	around 10	100	15	-	-	lyophilization	n.d.	DPPH IC_50_ 4.53 mg/mL	
*Cyprinus Carpio*	roe	alcalase	1.5:100	8	55	3	12.7	85-95	20	-	-	vacuum drying	n. d.	DPPH IC_50_ 1.151 mg/mL	Chalamaiah et al., 2015
	roe	pepsin	1.5:100	2	37	3	30	85-95	20	-	-	vacuum drying	n. d.	DPPH IC_50_ 2.25 mg/mL	
	roe	trypsin	1.5:100	8	37	3	19.5	85-95	20	-	-	vacuum drying	n. d.	DPPH IC_50_ 1.158 mg/mL	
*Octopus vulgaris*	muscle	protease from *Salaria basilica*	1:3	8	45	8	17.6	80	20	-	-	lyophilization	high MW peptides.	DPPH 53.29% (at 6 mg/mL)	Salem et al., 2017 [57]
	muscle	protease from *Bacillus subtilis* A26	1:3	8	45	8	21	80	20	-	-	lyophilization	highest level of peptides, with MW < 0.3 kDa	DPPH 75.1% (at 6 mg/mL)	
*Litopenaeus vannamei*	whole	Protamex	1:100	7	50	-	10 and 20	90	20	-	-	lyophilization	n. d.	ABTS about 48% (at 5.0 mg/mL)—values for DH 10% about 66% (at 5.0 mg/mL)–DH 20%	Latorres et al., 2017
	whole	alcalase	1:100	8	50	-	10 and 20	90	20	-	-	lyophilization	n. d.	ABTS about 68% (at 5.0 mg/mL)–values for DH 10% about 56% (at 5.0 mg/mL)–DH 20%	
*Gryllodes sigillatus*	whole	α-amylase, pepsin, pancreatin, and bile extract solution	4:100	-	37	1	-	100	5	-	-	lyophilization	MW ≤ 3.5 kDa	ABTS IC_50_ 15.24 µg/mL DPPH IC_50_ 17.97 µg/mL	Zielińska et al., 2017
*Tenebrio molitor*	whole	α-amylase, pepsin, pancreatin, and bile extract solution	4:100	-	37	1	-	100	5	-	-	lyophilization	MW ≤ 3.5 kDa	ABTS IC_50_ 24.31 µg/mL DPPH IC_50_ 85.55 µg/mL	
*Schistocerca gregaria*	whole	α-amylase, pepsin, pancreatin, and bile extract solution	4:100	-	37	1	-	100	5	-	-	lyophilization	MW ≤ 3.5 kDa	ABTS IC_50_ 12.1 µg/mL DPPH IC_50_ 88.81 µg/mL	
* Meleagris gallopavo *	muscle	flavourzyme	3:100	5.42	50.09	1.08	12.11	100	12	-	-	lyophilization	n. d.	ABTS 79.15% DPPH 88.33%	Wang et al., 2018
* Gallus gallus domesticus *	muscle	flavourzyme	3:100	5.42	50.09	1.08	12.86	100	12	-	-	lyophilization	n. d.	ABTS 78.23% DPPH 86.52%	
*Bovi*	muscle	flavourzyme	3:100	5.42	50.09	1.08	10.52	100	12	-	-	lyophilization	n.d.	ABTS 70.57% DPPH 80.15%	
* Camelus dromedarius *	milk	mixture of pepsin and pancreatin	-	5.3 then 7.5	37	4	-	100	10	-	-	lyophilization	LEEQQQTEDEQQDQL (MW: 1860.85 Da, LL-15); YLEELHRLNAGY (1477.63 Da, YY-11); RGLHPVPQ (903.04 Da, RQ-8)	ABTS IC_50_ < 0.01 mg/mL DPPH IC_50_ 0.01 mg/mL —for YY-11 ABTS IC_50_ 0.03 mg/mL DPPH IC_50_ 0.03 mg/mL —for LL-15 ABTS IC_50_ 0.07 mg/mL DPPH IC_50_ 0.06 mg/mL —for RQ-8	Homayouni-Tabrizi et al., 2017
* Camelus dromedarius *	milk	alcalase	1:100	8	50	6	15.5	100	10	-	-	lyophilization	n. d	DPPH about 19 µmol TE/g	Al-Shamsi et al., 2018
	milk	bromelain	1:100	7	50	6	23.8	100	10	-	-	lyophilization	n. d.	DPPH 23 µmol TE/g),	
	milk	papain	1:100	7	50	6	39.6	100	10	-	-	lyophilization	n. d	DPPH: 21 µmol TE/g	
*Buffalo*	milk	trypsin	1:100	7-9	37	3	-	90	15	-	-	lyophilization	RELEE, MEDNKQ, RELEEL, LQS, GNACF,	n.d.	Shazly et al., 2017
	milk	alcalase	1:100	5-7	55	3	-	90	15	-	-	lyophilization	PRG, TVA, TAAG, HCL, LLSLS	n.d.	
* Gallus gallus domesticus *	egg white	protease from *Y. lipolytica*	1:30	8	37	24	38	100	15	-	-	lyophilization	MW < 3 kDa	DPPH 0.21 μM Trolox_eq_/mg	Pokora et al., 2017
* Gallus gallus domesticus *	egg yolk	proteinase from *C. ficifolia*	1:7.52	8	37	4	-	100	15	-	-	lyophilization	LAPSLPGKPKPD	DPPH 6.03 μM Trolox_eq_/mg FRAP 296.07 μg Fe^2+^/mg	Zambrowicz et al., 2015 [58]

* increased radical scavenging activity in comparison to unhydrolyzed protein. n.d, not determined; PH, protein hydrolysates; **G**, Glycine; **P**, Proline; **A**, Alanine; **V**, Valine; **L**, Leucine; **I**, Isoleucine; **M**, Methionine; **C**, Cysteine; **F**, Phenylalanine; **Y**, Tyrosine; **W**, Tryptophan; **H**, Histidine; **K**—**R**, Arginine; **Q**, Glutamine; **N**, Asparagine; **E**, Glutamic Acid; **D**, Aspartic Acid; **S**, Serine; **T**, Threonine.

**Table 2:** Methods of obtaining protein hydrolysates of plant origin.

Source	Part of Source	Enzymatic Hydrolysis						Enzymatic Deactivation				Dehydration	MW of PH/ Peptides Sequence	Antioxidant Activity PH/Peptide	References
		Type of Enzyme	E/S Ratio	pH	Temp (°C)	Time (h)	DH (%)	Temp (°C)	Time (min)	pH	Reagent				
*Tetradesmus obliquus*	whole	alcalase	1:10	8	60	4	-	-	-	2	TFA	lyophilization	WPRGYFL, GPDRPKFLGPF, WYGPDRPKFL, SDWDRF	EC_50_ = 4.70 EC_50_ = 13.97 EC_50_ = 0.82 EC_50_ = 5.73	Montone et al., 2018
*Palmaria palmata*	whole	Corolase^®^ PP	1:100	7	50	4	-	90	20	-	-	lyophilization	SDITRPGGQM	ORAC: 152.43 [TE/μmol] FRAP: 21.23 [TE/μmol]	Harnedy et al., 2017
*Fucus spirali*	whole	cellulase then bromelain	1:100	4.5 then 7	50 then 37	20	-	100	10	-	-	lyophilization	MW F1: <1 kDa F2: 1–3 kDa F3: >3 kDa	FRAP: F1: 86.03 F2: 32.73 F3: 80.50	Paiva et al., 2017
*Spirulina* sp.	whole	pepsin then trypsin and α-chymotrypsin	1:100	2.2 then 6.5	37	2 then 2.5	-	100	10	-	-	lyophilization	TMEPGKP	IC_50_ 0.1 mg/ml	Heo et al., 2017
*Schizochytrium* sp.	whole	alcalase then flavourzyme	10:100 then 12.5:100	9 then 6.7	50	6 then 8	8.37 then 21.48	100	10	-	-	lyophilization	MW <3 kDa >3 kDa	DPPH: IC_50_ 350 μg/mL ABTS: IC_50_ 17.5 μg/mL, for: MW <3 kDa	Cai et al., 2017
*Agaricus bisporus*	fruiting bodies	alcalase	2.5:100	9	50	4	about 18	100	15	-	-	lyophilization	MW <1 kDa 1–3 k Da 3–5 kDa 5–10 kDa	DPPH (MW < 1; 1–3; 3–5; 5–10 kDa) about EC_50_ 0.64; 0.32; 0.64; 0.61	Kimatu et al., 2017
	fruiting bodies	pancreatin	2.5:100	7.5	37	4	about 12.5	100	15	-	-	lyophilization,	MW <1 kDa 1–3 kDa 3–5 kDa 5–10 kDa	DPPH (MW < 1; 1–3; 3–5; 5–10 kDa) about EC_50_ 0.64; 0.13; 0.64; 0.21)	
	fruiting bodies	flavourzyme,	2.5:100	7	50	4	about 3	100	15	-	-	lyophilization	MW <1 kDa 1–3 k Da 3–5 kDa 5–10 kDa	DPPH (MW < 1; 1–3; 3–5; 5–10 kDa) about EC_50_ 0.64; 0.25; 0.64; 0.30)	
	fruiting bodies	alcalase-pancreatin	2.5:100	9.0 then 7.5	50 then 37	2 then 2	about 18.5	100	15	-	-	lyophilization	MW <1 kDa 1–3 kDa 3–5 kDa 5–10 kDa	DPPH (MW < 1; 1–3; 3–5; 5–10 kDa) about EC_50_ 0.64; 0.42; 0.64; 0.24)	
	fruiting bodies	alcalase-flavourzyme	2.5:100	9.0 then 7.0	50	2 then 2	about 16	100	15	-	-	lyophilization	MW <1 kDa 1–3 kDa 3–5 kDa 5–10 kDa	DPPH (MW < 1; 1–3; 3–5; 5–10 kDa) about EC_50_ 0.64; 0.38; 0.64; 0.16)	
*Zingiberaceae*	rhizome	pepsin then pancreatin	1:20	2.5 then 7.5	37	3 then 3	-	80	20	-	-	lyophilization	MW 12.4–12.8 kDa	IC_50_ 41. 78 µg/mL	Inthuwanarud et al., 2016
*Cucurbit* *moschata*	seed	trypsin	1:50	7.5	37	4	-	heated in water bath	20	-	-	lyophilization	n. d.	DPPH: IC_50_ 49.3 µg/mL ABTS: IC_50_ 142.3 µg/mL	Dash et al., 2017
*Citrullus lanatus*	seed	trypsin	1:50	7.5	37	4	-	heated in water bath	20	-	-	lyophilization	n. d.	DPPH: IC_50_=80.3 µg/Ml ABTS: IC_50_=179 µg/mL	
*Lagenaria siceraria*	seed	trypsin	1:50	7.5	37	4	-	heated in water bath	20	-	-	lyophilization	n. d.	DPPH: IC_50_ 46 µg/mL ABTS: IC_50_ 108 µg/mL	
Citrullus lanatus	seed	pepsin	1:100	2.2	37	5	19.38	95–100	15	-	-	lyophilization	n. d.	IC_50_ 2.41 µg/mL	Arise et al., 2016 [72]
	seed	trypsin	1:100	8	37	5	26.26	95–100	15	-	-	lyophilization	n. d.	IC_50_ 2.82 µg/mL	
	seed	alcalase	1:100	8	60	5	13.16	95–100	15	-	-	lyophilization	n. d.	IC_50_ 3.20 µg/mL	
*Silybum marianum*	seed	neutrase	1:60	7	55	2	-	100	10	-	-	lyophilization	MW <1 kDa 1–3 kDa 3–10 kDa ≥10 kDa	TAOC 0.89 U/mg (at 800 mg/kg)	Zhu et al., 2017 [73]
*Mungfaba*	defatted mungbean meal	bromelain	5, 10, 15, 20:100	6	50	6, 12, 18, 24	50.4	95	15	-	-	evaporation	MW <10 kDa	DPPH and ABTS 80; 90%	Sonklin et al., 2018
*Vigna radiata*	seed	alcalase	20 × 10^4^ U g^−1^	9.5	60	2.5	61.5	100	10	-	-	lyophilization	n.d.	DPPH and ABTS: IC_50_ = 0.77 and 0.78 µg/mL,	Gupta et al., 2018
	seed	trypsin	25 × 10^4^ U g^−1^	8	37	3.5	46.4	100	10	-	-	lyophilization	n.d.	IC_50_ about 1.3 µg/mL	
*Phaseolus vulgaris*	pod	pepsin	1:20	2	37	2	-	100	5	-	-	lyophilization	n. d.	DPPH and ABTS 46.12%; 92.32%—hydrolysates obtained from heat treated beans	Karaś et al., 2014
*Phaseolus vulgaris*	seed	alcalase	1:20	2	50	7	11.5	90	10	-	-	lyophilization	n. d.	higher ABTS scavening activity for alcalase treated protein	Evangelho et al., 2017 [74]
	seed	pepsin	1:20	2	37	2	27.09	90	10	-	-	lyophilization	n. d.		
*Vigna subterranea*	seed	alcalase	4:100	7	55	24	38	95	5	-	-	lyophilization	hydrolysates produced using trypsin contained higher large-size peptides (>3.5 kDa) compared to the other hydrolysates	DPPH 0.781 µg trolox eq./mg	Mune et al., 2018
	seed	trypsin	1:100	7	55	24	22	95	5	-	-	lyophilization		DPPH 5.52 µg trolox eq./mg	
	seed	thermolysin	1:100	8	70	24	27.5	95	5	-	-	lyophilization		DPPH 1.323 µg trolox eq./mg)	
*Glycine max*	defatted soy flour	peptidases from latex of *Maclura pomifera* fruits	1:10	8.0	45	3	36.2	100	7	-	-	-	Theoretical sequences of peptides (D)LDIFLSSVDINEGAL(L) (I)PAAYPFVVNATSNLNFLA(F) R)FQTLFKNQYGHVRVLQRFN(K) (Y)NLQSGDALRVPAGTTFYV(V)	IC_50_ 31.6 µg/mL, ABTS 157.6 µg trolox eq./mg ORAC 176.9 µm TE/g	Jara et al., 2018 [75]
*Glycine max*	defatted soy flakes	Corolase PP	3:100	7.5	50	5	about 26 for 80 MPa, about 27 for 100 MPa, about 28 for 120 MPa, about 29 for 200 MPa, about 31 for 300 MPa	100	10	-	-	lyophilization	MW < 3 kDa	ABTS 30.6%	Guan et al., 2018
*Voandzeia subterranea*	seed	alcalase	1:100	8	50	4	-	90	15	4	2M HCl	lyophilization	n.d.	EC_50_ about 25 μg/mL	Arise et al., 2017 [76]
	seed	pepsin	1:100	2	37	4	-	90	15	4	2M NaOH	lyophilization		EC_50_ about 22 μg/mL	
	seed	trypsin	1:100	8	37	4	-	90	15	4	2M HCl	lyophilization		EC_50_ 22 μg/mL	
*Frumentum*	corn gluten meal	alcalase	9.13:100	8.6	50	2.5	-	100	10	-	-	lyophilization	AGIPM, AGLPM, HALGA, and HAIGA H1: MW < 1 kDa) H2: 10 kDa < MW < 30 kDa	DPPH H1: 66.89% H2: 71.49%	Jiang et al., 2018
*Rice furfures*	defatted rice bran	pepsin then trypsin	1:100	1.5 then 7	37	2 then 2	-	95	10	-	-	lyophilization	F1: MW < 3 kDa, F2: MW 3–5 kDa, and F3: MW 5–10 kDa)	F1;F2;F3 DPPH 66.25; 58.57; 43.98 µmoL Trolox equivalent/g ABTS 425.81; 430.12; 403.28 µmoL Trolox equivalent/g	Phongthai et al., 2018
*Pennisetum glaucum*	seed	trypsin	1:100	6.5	37	3	-	80	20	-	-	lyophilization	SDRDLLGPNNQYLPK	DPPH 67.66%, ABTS 78.81%, Fe^2+^ chelating ability 51.20%,	Agrawal et al., 2016 [77]
*Amaranthus hypochondriacus*	seed	endogenous aspartic protease	-	2	40	16	5.3	85	10	-	-	lyophilization	n.d.	hydrolysate ORAC IC_50_ 0.058 ABTS IC_50_ 2.1 mg/mL	Sabbione et al., 2016 [78]
*Cannabis sativa*	seed	pepsin	1:50	2	37	16	19.7	95	10	-	-	-	Pepsin hydrolysis—high number of peptides (1000–1500; 2000–2500 Da)	n.d.	Aiello et al., 2017
	seed	trypsin	1:50	8	37	16	46.6	95	10	-	-	-		n.d.	
	seed	pancreatin	1:50	8	37	16	47.5	95	10	-	-	-		n.d.	
	seed	pepsin then the mixture of trypsin and pancreatin	1:20 then 1:25	2 then 8.5	37	2 then 4	34	95	10	-	-	-		n.d.	
*Tinospora cordifolia*	stem	papain	1:100	6.8	37	2	-	100	0.1	-	-	lyophilization	VLYSTPVKMWEPGR; VITVVATAGSETMR; HIGININSR	SRCA 64.15% at 0.0125 mg/mL	Pachaiappan et al., 2018
	stem	pepsin	1:3.33	2.2	37	2	-	-	-	8	Na_2_CO_3_	lyophilization	n.d.	DPPH about 52%—for trypsin hydrolysate	
	stem	trypsin and α-chymotrypsin	1:5	7.8 then 8	37	3	-	100	0.1	-	-	lyophilization	n.d.	DPPH 79.04%—for trypsin hydrolysate after 30 min digestion	
*Moringa oleifera*	seed	trypsin	1:5	7.8	37	2.5 and 5	-	80	10	-	-	lyophilization	Peptide fraction > 10 kDa	ABTS 24.74; 32.81%	Garza et al., 2017
	seed	chymotrypsin	1:5	7.8	37	2.5 and 5	-	80	10	-	-	lyophilization	Peptide fraction > 10 kDa	ABTS 35.32; 37.87%	
	seed	pepsin–trypsin	1:5	2 then 7.8	37	2.5 and 5	-	80	10	-	-	lyophilization	Peptide fraction > 10 kDa	ABTS 29.15; 29.30%	
*Limonia acidissima*	seed	pepsin	2.5:100	2	37	42.41	39.82	100	10	-	-	air-drying	n.d	DPPH 32.94% ABTS 88.18%	Sonawane et al., 2017
*Juglans regia* L.	defatted walnut meal	pancreatin and viscozyme L	0.8:100	7	55	16	6.6	95	15	-	-	lyophilization	n.d	ORAC 1752.98 μmol TE/g ABTS 237.94 μmol TE/g) at 0.4 mg/mL	Li et al., 2017

n. d., not determined; PH, protein hydrolysates; SRCA, superoxide radical scavenging activity; TAOC, total antioxidant capacity; **G**, Glycine; **P**, Proline; **A**, Alanine; **V**, Valine; **L**, Leucine; **I**, Isoleucine; **M**, Methionine; **C**, Cysteine; **F**, Phenylalanine; **Y**, Tyrosine; **W**, Tryptophan; **H**, Histidine; **K**—**R**, Arginine; **Q**, Glutamine; **N**, Asparagine; **E**, Glutamic Acid; **D**, Aspartic Acid; **S**, Serine; **T**, Threonine.

## Data Availability

The analyzed publications are available from the authors.
